# Editorial: Explainable Artificial Intelligence and Neuroscience: Cross-Disciplinary Perspectives

**DOI:** 10.3389/fnbot.2021.731733

**Published:** 2021-08-03

**Authors:** Jeffrey L. Krichmar, James Leland Olds, Juan V. Sanchez-Andres, Huajin Tang

**Affiliations:** ^1^Department of Cognitive Sciences, University of California, Irvine, Irvine, CA, United States; ^2^Schar School of Public Policy, George Mason University, Arlington, VA, United States; ^3^Department of Medicine, University of Jaume I, Castellón de La Plana, Spain; ^4^College of Computer Science and Technology, Zhejiang University, Hangzhou, China

**Keywords:** explainable AI, neuroscience, computational neuroscience, neural networks, machine learning

In this Research Topic, we convened computational neuroscientists, roboticists, and AI researchers to consider the problem of explainable AI from different perspectives. Though deep learning is the main pillar of current AI techniques and is ubiquitous in basic science and real-world applications, it is also constrained by AI researchers for its black-box problem: it is easy to fool, and it also cannot explain how it makes a prediction or decision. Therefore, the significance of creating transparent, explainable AI, should not be underestimated. In particular, we are interested in leveraging insights from neurobiology that might be useful for novel approaches in AI, and techniques for analyzing artificial neural networks that might be applied, in turn, to neuroscience.

As Artificial Intelligence (AI) becomes more pervasive and intelligent, its failures will become more salient. This is not a paradox. In both the case of biological brains and AI, intelligence involves decision-making using data that are noisy and often ambiguously labeled. Input data can also be incorrect due to faulty sensors. Moreover, during the skill acquisition process, failure is required to learn.

When human intelligence fails with significant consequence, accepted best practice requires that we look for root causes, adapt, and learn from our mistakes. When AI fails, we increasingly will require an explanation for what went wrong. Many AI devices being deployed are making human biased decisions that could turn out to be wrong. The actual consequence is to determine responsibility both in the legal and economic respects. It is unlikely that AI can be free to be broadly applied in real contexts if liability assignment is not clearly defined. Then, an unexpected legal frontier may appear with interesting implications. As with human testimonies, an explanation for how an AI decision came to fruition is challenging. In all cases, biological and AI decision-making is distributed across a large number of nodes (or neurons) that each contributes to the final product to some varying degree. We believe neuroscience methods may help to clarify decision-making in both natural and artificial systems.

Computational neuroscientists construct models to better understand the inner workings of the brain. In a broad sense, this approach also applies to AI. However, the goal is oftentimes to make predictions of how brain activity can lead to behavior. Roboticists design control systems to perform some function. From an observer point of view, the robot's behavior can provide explanations of how the system is making decisions. In this Research Topic, several of the papers show how this activity and behavior leads to explainability. Other papers take inspiration from biology to make neural networks more interpretable. In sum, the seven papers in this Research Topic bridge the gaps between AI and neurobiology.

One of the most exciting areas of neuroscience is the neural context of navigation or the so-called cognitive map. The discovery of place cells, head direction cells, and grid cells have intrigued neuroscientists and modelers alike (Derdikman and Moser, 2010). These spatial neural representations can be interpreted to know where the animal has been, where it is now and where it intends to go. Although many of the findings have been observed in rats navigating mazes, the brain areas involved have homologs to human brain areas important for not only navigation, but also human learning and memory. Two of the papers in the Research Topic introduced models of rodent navigation (Wang et al.; Yu et al.). In Wang et al., the authors showed how combining multi-scale grid cell neural networks with place cells and a hierarchical vision system can lead to the creation of cognitive maps. This neurobiologically inspired framework was tested on a robot and shown to navigate successfully. Although not specifically designed for explainability, the maps generated by the robot exploration and the robot's trajectories are interpretable and shed light on how successful navigation is achieved. In Yu et al., the authors extend the well-known neurobiologically inspired RatSLAM algorithm (Milford et al., 2016), with saliency maps. In computational neuroscience, saliency maps can predict where a person will focus attention (Itti and Koch, 2001). Furthermore, saliency maps have been applied in AI to explain what features the neural network used in making a decision (Greydanus et al., 2018). It is interesting that this addition also improved the performance of RatSLAM.

A couple of approaches in the Research Topic demonstrate state-of-the art performance while improving explainability. In one paper, a symbolic inference engine is integrated with a traditional machine learning approach (Hernández-Orozco et al.). The authors show that this approach could be robust in preventing attacks. Furthermore, introducing symbolic computation can lead to interpretability. In Dick et al., the authors propose that using statistical tests to estimate predictive models can overcome catastrophic forgetting. The probability distributions of predictive models provide an explainable method to assess and justify the model's beliefs about the current environment.

Donald Hebb postulated that cellular assemblies are an important feature of neural processing (Hebb, 1949). The Biologically Enhanced Artificial Neuronal (BEAN) assembly applies this idea to a neural network that shows state of the art performance on several AI and neural network benchmarks (Gao et al.). During learning, functional cell assemblies form that are themselves interpretable.

Two papers in the Research Topic reviewed neurobiologically inspired approaches that could lead to explainability. In (Chen et al.), the authors proposed that neurorobotics can provide explanations for how neural networks lead to behavior. Observing robot behavior can be thought of as a form of neuroethology. In contrast to neuroethology work in animals, the neuroroboticist has full access to the artificial brain during behavior, which leads to further explainability. In Grossberg reviews the large body of work on Adaptive Resonance Theory (ART). Grossberg shows how ART can explain a wide range of brain phenomena ranging from motor systems to sensory systems to emotion.

In conclusion, the Research Topic on Explainable Artificial Intelligence and Neuroscience is an important step toward improving AI by looking to biology for inspiration. Because these models are accessible and interpretable, they can further our understanding of the brain and lead to the design of neural networks that open the black box. We further hope that some of these ideas can inspire neuroscientists to achieve better interpretations of their data, as well as lead to a tighter collaboration between AI, biology, computer science, and engineering researchers.

## Author Contributions

All authors listed have made a substantial, direct and intellectual contribution to the work, and approved it for publication.

## Conflict of Interest

The authors declare that the research was conducted in the absence of any commercial or financial relationships that could be construed as a potential conflict of interest.

## Publisher's Note

All claims expressed in this article are solely those of the authors and do not necessarily represent those of their affiliated organizations, or those of the publisher, the editors and the reviewers. Any product that may be evaluated in this article, or claim that may be made by its manufacturer, is not guaranteed or endorsed by the publisher.
